# Hospital Sunday

**Published:** 1887-06-18

**Authors:** 


### HOSPITAL SUNDAY.

*June 19th*, 1887.

Long have the Sabbath bells,
O'er England's hills and dells,
Rung out their holy message soft and clear,—
Rung over all the land
As tho' one mighty hand,
Moved by one impulse, peals them far and near.

But tho' the bells to-day
Would summon hearts to pray,
They seem to strive to tell us something more,
And on our spirits fall
The old familiar call,
Fraught with a deeper meaning than of yore.

Is discord in the air?
What is the sorrow there
hat, all dissatisfied, they strive to tell?
At last an answer clear
Falls upon every ear,
Like the sad measure of a funeral knell:
Ye Christian worshippers arise,
And listen, listen to the story from the skies.

"On each town and on each city
God and angels look with pity,
On the fever-crowded alleys
Ye ne'er dream of in your valleys—
Where the children, with white faces,
Walk with slow and languid paces;
Where the sick and weary mothers
Bear their load the same as others,
And, in place of gentle nursing,
Weakness oft is met with cursing
Where the maimed and halt are lying
And for succour vainly crying—
Sick, diseased, and weak, or ailing,
Up to heaven ascends their wailing."

Then in sudden anger
Seem the bells to clangour,
As our hearts, unanswering, still attend,
And with startled eyes
We gaze toward the skies,
List'ning, waiting for the solemn strains to end.

"Ye, whose poorer brethren languish,
How can ye behold their anguish?
And, with hearts unmoved to pity,
Leave them in each crowded city?
Will ye pass them by unheeding
All their ceaseless, bitter pleading?
Every town around you lying,
Every alley swells the crying:—
'Take our sick, with skill and tending,
Air and space and sunshine blending,
Oh! relieve their labouring breath,
Save them from this living death.
Here we have no open spaces,
Poverty her finger traces
All around, while weak ones cry
For nourishment we cannot buy;
And, when bread is hard to win,
Hard it is to keep from sin.'"

Gently the bells again
Renew a softer strain,
And now with earnest pleading seem to ring,
Slow beats each silent heart,
While "tears unbidden start"
And fall, for very pity, as they sing.

"There are buildings high and spacious,
And the workers there are gracious;
Oh! their looks of gentle pity
When the toilers from each city
Bear a sister or a brother,
Or a frail and dying mother,
And implore them to receive them,
And for mercy to relieve them.
Few, for want of means, are taken,
Many, many, left forsaken.
Ye, who of your Master's treasure
On the earth have reap'd full measure,
Yours it is to carry gladness
To those homes of gloom and sadness;

Now, the story ending
With one peal ascending,
Half in final warning, half in pity,
Then float the chimes away,
Yet seem awhile to stay,
Like answers to the cry from every city.

Then let us, one and all,
Rise nobly to the call,
And own our solemn duty of *relieving*,
With generous heart and hand,
The toilers of our land,
And feel more joy in *giving* than *receiving*.

Can Christian brethren dare
To bend the knee in prayer,
Unheeding those sad pleadings of the bells?
Nay, towards the fainting bands
We stretch our willing hands,
As from each voice responsive pity swells.

"Hospitals, with half-closed portals,
Open wide to struggling mortals;—
From each crowded city's din
Take the weak and dying in.
Ours the hands to carry gladness
To those homes of gloom and sadness;
Theirs the blessing of receiving,—
Ours the glory of relieving.

Men of science! gentle nurses!
Toiling on against reverses,
While your tender hands can labour,
Raising up each weary neighbour,
All your lives 'midst sickness living—
How shall *we* be slow in giving?—
How—if giving all—could we
Match such perfect charity?"

Slowly our voices cease,
And all within is peace,
As in God's House the organ's hymn floats clear,
Then rises loud and free,
Like strains of Jubilee,
Crowning the chiefest glory of this glorious year.

ANON.

